# Small Bowel Obstruction Secondary to an Expelled Enterolith From Jejunal Diverticulum: A Rare Entity

**DOI:** 10.1155/cris/2637594

**Published:** 2025-12-01

**Authors:** Muhammed Ali Zishan, Hannah Tang, Bettina Schulze

**Affiliations:** Department of Surgery, Mackay Base Hospital, Mackay, Queensland, Australia

## Abstract

We describe a rare case of a mechanical small bowel obstruction secondary to multiple enteroliths expelled from underlying jejunal diverticular disease. A 59-year-old male, without any past surgical history presented to a regional hospital in Queensland with symptoms consistent with an acute small bowel obstruction. A CT scan performed on arrival confirmed the diagnosis of a small bowel obstruction with a transition point at the level of mid small bowel however the aetiology was not radiologically apparent. He proceeded to an exploratory laparotomy which revealed at least three intraluminal enteroliths, one of which was impacted within the mid jejunum resulting in the bowel obstruction. These enteroliths were all milked distally and successfully retrieved via an enterotomy in a healthy segment of distal ileum. He was also noted intraoperatively to have extensive proximal jejunal diverticular disease as the likely source of his dislodged enteroliths. Retrospectively, his CT scan could be correlated to his intraoperative findings, bringing to light this rare phenomenon which has been documented only in a handful of published cases within surgical and gastroenterology literature.

## 1. Introduction

Jejunal diverticulosis is relatively rare with a reported incidence in literature between 0.02% and 7.1% of the population [1]. They are largely asymptomatic, more common in the elderly with a slight male predominance [2]. Cases of jejunal diverticulosis can occasionally be complicated by acute diverticulitis, haemorrhage, intussusception, volvulus, formation of strictures/adhesions and even small bowel obstruction secondary to enterolithiasis [3]. Intraluminal enteroliths also known as primary enteroliths develop in regions of intestinal stasis, such as within diverticulae, where the shift in pH favours precipitation of choleic acids often in conjunction with undigested food [1]. Occasionally, these enteroliths can become dislodged from within the diverticulum where it can lead to a mechanical obstruction downstream in a phenomenon known as ‘enterolith ileus' [3] as seen in this case. Whilst an obstruction secondary to enterolithiasis may be managed conservatively; failing to proceed with this approach or in cases complicated by localised perforation, often necessitate surgical intervention and several surgical techniques have been described in literature, including manual lysis of the enterolith and subsequent milking into distal bowel, enterotomy and extraction of the enterolith and occasionally resection and subsequent anastomosis of the bowel containing the enterolith [1].

## 2. Case Presentation

A 59-year-old male presented to the local emergency department in a regional hospital in Queensland with recurrent colicky central abdominal pain for the past 3 days with associated nausea and multiple episodes of vomiting. He was still passing flatus at the time of presentation having last opened his bowels the day prior. He was an active smoker with a significant medical history of type 2 diabetes which was well controlled on metformin. He did not have any prior surgical history.

On examination, he was hemodynamically stable and afebrile, appearing clinically well. His abdomen was mildly distended and tender over the right lower quadrant and right lumbar region without any features of peritonism with audible bowel sounds. Incidentally a left completely reducible fat containing inguinal hernia was noted during examination which was felt to be unrelated to his clinical picture.

**Figure 1:**
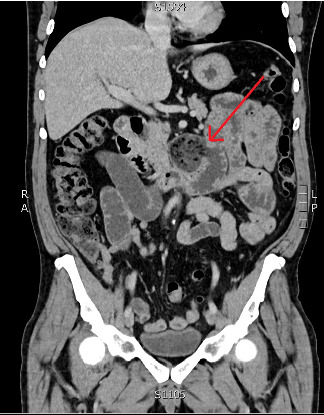
CT coronal view with arrow pointing to a large proximal jejunal diverticulum containing mixed densities without any evidence of inflammation.

**Figure 2:**
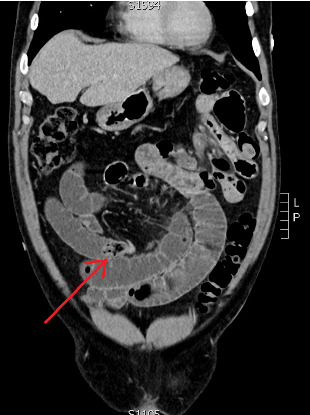
CT coronal view with the arrow marking the transition point of the small bowel obstruction with the enterolith visible just proximal to the collapsed loop of small bowel distally.

Initial bloodwork revealed raised inflammatory markers with WCC of 15 × 10^9^/L, neutrophilia of 13 × 10^9^/L and a C-reactive protein of 95 mg/L. His renal function, liver function tests and venous blood gas were all normal. He proceeded to an urgent CT scan of his abdomen and pelvis with intravenous contrast as seen Figures 1 and 2, which confirmed the diagnosis of small bowel obstruction with a transition point in the mid small bowel towards the right side of his abdomen. There was no free fluid nor any lymphadenopathy. Of note, his gall bladder was collapsed and did not contain any radio-opaque calculi, nor were there any biliary tree abnormalities. The radiologist offered the differentials to his aetiology being either adhesion related or possibly an internal hernia, both of which were unlikely in a patient with a virgin abdomen.

**Figure 3:**
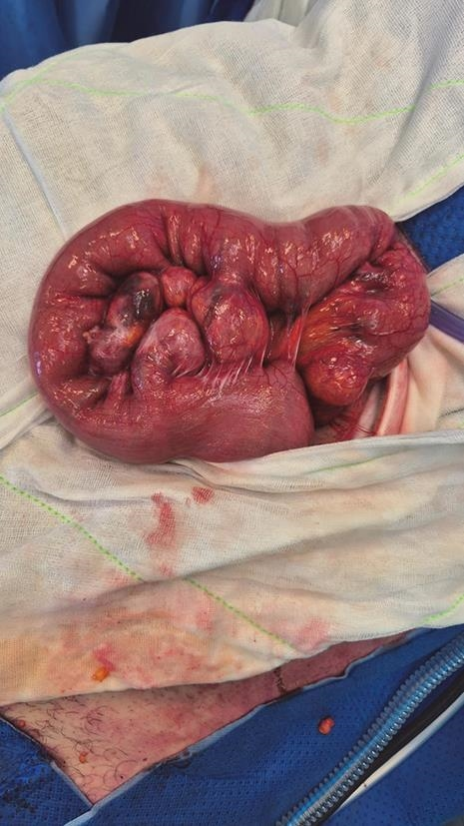
Intra operative view of a loop of proximal jejunum roughly 30 cm from the DJ flexure containing multiple large diverticula some of which contained evolving enteroliths but not obstructing the lumen. These were the likely culprit from which the enteroliths had dislodged resulting in a distal small bowel obstruction.

Given the clinical picture a trial of conservative management was attempted with nasogastric tube decompression and nil by mouth status, however it was felt that he was likely to need an operative intervention. He was assessed the following morning and given non-resolution of his bowel obstruction was taken to theatre for a diagnostic laparoscopy ± laparotomy ± bowel resection. On entering his abdomen laparoscopically, a moderate volume of serous fluid was encountered which were all suctioned out. The entirety of his small bowel was found to be healthy but distended upto a point in the mid jejunum where there was an impression of an intraluminal mobile mass suspicious for a bezoar. Two other similar masses were identified more proximally. Given the unclear nature of these masses and to avoid iatrogenic bowel injury, a mini laparotomy was performed to deliver the small bowel for further inspection. These lumps were easily milked distally into a region of unobstructed healthy appearing small bowel where a transverse enterotomy was made to deliver the calcified stone like masses which were sent for histology. On evacuation of these stones, the enterotomy was closed in a double layer ensuring the lumen remained patent. Further examination upstream revealed multiple proximal jejunal diverticulum within the mesentery roughly 30 cm from the duodenojejunal (DJ) flexure as seen in Figure 3; the likely culprit behind the stones found distally. Some of these diverticulae on palpation contained smaller stones, however given the non-obstructive nature of these enteroliths and the proximity to the DJ flexure, it was left undisturbed without resection.

The laparotomy wound was subsequently closed with rectus sheath catheters for analgesia. The patient progressed well on the ward, passing flatus on Day 1 post op and eventually opening bowels satisfactorily on Day 3. He was discharged on Day 4, following a dietitian review to ensure he remains on a lifelong low residue diet given the possibility of a recurrence in the future. He was informed about his underlying disease and was advised to represent if he developed any further episodes of bowel obstruction in the future.

## 3. Discussion

Diverticular disease is a common finding within the large bowel however less so within the small bowel. When present, it usually affects the duodenum in nearly 60%–70% of the cases, with 20%–25% within the jejunum and just 10% in the ileal region [4]. Small bowel diverticula are a type of false diverticulosis as they do not contain all the tissue layers and its pathogenesis, while not entirely clear, is felt likely to be secondary to a combination of gut dysmotility with subsequent higher intraluminal pressures resulting in mucosal herniation [4]. The penetrating mesenteric vessels along the mesenteric border of the small bowel create potential areas of weakness within the muscularis propria resulting in the mucosal herniation seen in small bowel diverticular disease [4].

Whilst majority of patients with jejunal diverticulosis are asymptomatic, 30% of these become symptomatic and about 10% develop complications which require surgical intervention [1] as seen in this case. Enteroliths have been postulated to develop within these diverticulae either in a de-novo fashion via the precipitation of bile salts within the slightly less acidic environment of the diverticulae or via the accumulation of indigestible food matter/bezoar acting as a nidus for stone formation [1]. The pathogenesis of these endogenous enteroliths termed ‘primary enteroliths' are quite different to ‘secondary enteroliths' which require fistulisation into the gastrointestinal tract such as those seen with a gallstone ileus [3]. These primary enteroliths can occasionally cause local perforation by pressure necrosis or they may obstruct via direct encroachment of the adjacent lumen or spontaneously dislodge resulting in distal impaction as seen in this case [3].

The diagnosis of an enterolith causing an obstruction or an ‘enterolith ileus', can be made either during surgery or careful analysis of pre-operative imaging, which can often be missed due to its obscure nature [4], but could be retrospectively correlated as in this case. This highlights the need to raise awareness of this phenomenon as the presence of this radiological finding should prompt the consideration of an enterolith ileus as a possible differential.

Whilst the definitive management for jejunal diverticular disease would involve resection of the affected segment of bowel to avoid recurrence, this is often not possible due to the risks associated with a proximal anastomosis in an obstructed segment of bowel as seen in this case and in a similar case published by Hoskin et al. [5]. The risk of recurrent small bowel obstruction secondary to enterolithiasis is unknown in published literature, however, Lee and Menezes [6] did report one such case occurring 7 months following enterotomy and extraction of enterolith. The treatment of choice is manual or instrument fragmentation of the enterolith and milking it distally into the colon which carries a nearly 50% success rate based on prior studies [1] however, an enterotomy is occasionally required for a safer extraction as in this case due to the presence of multiple large stone -like enteroliths. In cases of jejunal diverticulitis complicated by perforation, resection and subsequent anastomosis is warranted which may be done either via an open approach/laparotomy or even laparoscopically as described by Cui and Mehanna [7].

## Data Availability

The data that support the findings of this study are available on request from the corresponding author. The data are not publicly available due to privacy or ethical restrictions.
